# The potential for clinical pharmacists to support older people with dementia in the community: A qualitative interview study

**DOI:** 10.1002/bcp.70160

**Published:** 2025-07-08

**Authors:** Alice Burnand, Abi Woodward, Kumud Kantilal, Cini Bhanu, Yogini Jani, Jill Manthorpe, Mine Orlu, Greta Rait, Madiha Sajid, Kritika Samsi, Victoria Vickerstaff, Jane Ward, Jane Wilcock, Nathan Davies

**Affiliations:** ^1^ Research Department of Primary Care and Population Health Centre for Ageing Population Studies, University College London London UK; ^2^ Centre for Psychiatry and Mental Health, Wolfson Institute of Population Health, QMUL London UK; ^3^ Research Department of Practice and Policy School of Pharmacy, University College London London UK; ^4^ Centre for Medicines Optimisation Research and Education University College London Hospitals NHS Foundation London UK; ^5^ NIHR Policy Research Unit in Health & Social Care Workforce, King's College London London UK; ^6^ NIHR Applied Research Collaboration (ARC) South London, King's College London London UK; ^7^ Research Department of Pharmaceutics UCL School of Pharmacy, University College London London UK; ^8^ Research Department of Primary Care and Population Health PRIMENT Clinical Trials Unit, University College London London UK; ^9^ Centre for Evaluation and Methods, Wolfson Institute of Population Health, QMUL London UK; ^10^ Patient and Public Involvement member UK

**Keywords:** clinical pharmacists, dementia, family carers, primary care

## Abstract

**Aims:**

The prevalence of multiple long‐term health conditions including dementia is rising globally. Managing dementia presents significant challenges for healthcare providers. Clinical pharmacists, with expertise in medication management, have emerged as valuable members of the primary care team. However, there is a need for further research to understand their specific role and experiences in supporting people with dementia. The aim of this paper was to explore the views and experiences of primary care based clinical pharmacists in supporting people with dementia in the community.

**Methods:**

We conducted 13 semistructured interviews with primary care clinical pharmacists in England in 2023–2024. Data were analysed using reflexive thematic analysis.

**Results:**

Three overarching themes were developed from the data: (i) holistic care beyond medication management; (ii) the integral role and capacity of clinical pharmacists in the healthcare team and system when delivering dementia care; and (iii) building expertise in dementia care and defining the role of a clinical pharmacist.

**Conclusion:**

Clinical pharmacist roles have the potential to extend beyond medication management to deliver holistic support, as part of primary care based multidisciplinary teams and take a proactive approach to care for people with dementia. Findings highlight the importance of confidence, training and a supportive workplace environment for clinical pharmacists to effectively contribute to dementia care.

**What is already known about this subject**

- Clinical pharmacists are increasingly providing direct patient care.
- Clinical pharmacists contribute to patient safety due to their expertise in medication management.

**What this study adds**

- An increased understanding of the role of clinical pharmacists in supporting older people with dementia and their carers.
- Their role extends beyond medication management to support with holistic care.

## 1 INTRODUCTION

Over 982 000 people are living with dementia in the UK,^1^ with their healthcare mainly managed by a primary care team, typically led by their General Practitioner (GP).^2^ There is currently no cure for dementia and so the focus of care is on symptom management and providing high‐quality care through to end of life. On average, people with dementia have 4.6 additional long‐term conditions^3^ and are prescribed 5 medications.^4^, ^5^ Managing medication for people with dementia is particularly challenging due to difficulties with memory, understanding and communication.^6^ Collaborative, multidisciplinary care services can improve the management of multiple long‐term conditions (MLTCs) which many with dementia experience, but research is needed to explore how to facilitate effective health and care support for people with MLTCs and dementia. Given the challenge of MLTCs and dementia on healthcare services worldwide, providing high‐quality patient care is paramount.

Pharmacists are increasingly providing direct patient care,^7^ with their role in general practice becoming more clinically oriented.^8^, ^9^, ^10^ This area of practice and research has developed worldwide, due to the identified need to focus on ensuring medication optimisation and adherence to improve patient outcomes.^11^ Some countries (e.g. Canada, USA, Australia and the UK) have been building up comprehensive clinical pharmacy services to support direct patient care and medicines management.^11^, ^12^ Nearly a decade ago, NHS England launched a Clinical Pharmacists in General Practice pilot scheme. This demonstrated that clinical pharmacists have the potential to contribute to patient safety by using their expertise in medicines and prescribing to help patients with long‐term conditions, including promoting healthy lifestyles.^13^

Although there is growing understanding of the potential role of clinical pharmacists in primary care,^14^, ^15^, ^16^, ^17^, ^18^ less is known about their specific role and experiences in working with older people with dementia and their carers. Most UK pharmacy research within dementia care has focused on community pharmacists, whose roles and responsibilities differ from those of clinical pharmacists in primary care.^18^, ^19^ Community pharmacy is primarily involved in dispensing in retail pharmacy settings^18^ in comparison to clinical pharmacists who undertake prescribing and medication reviews and deprescribing (reducing or stopping medication).^19^ In addition, little is known about how clinical pharmacists feel about providing care for people with dementia and any specific training needs.^20^

### 1.1 Aims and objectives

This study aimed to explore clinical pharmacists' experiences of supporting people with dementia, identifying key responsibilities, services and training needs when based in England's primary care services.

## 2 METHODS

### 2.1 Research design

This research employed a qualitative exploratory study design, utilising semistructured interviews with clinical pharmacists working in NHS primary care in England. England was chosen following the implementation of the Clinical Pharmacists in General Practice pilot, and efforts by NHS England to increase numbers of clinical pharmacists in England.^21^, ^22^ Similar schemes and clinical pharmacists are also active within the other home nations in the UK but were not in scope for this study. Interviews were analysed using reflexive thematic analysis.^23^ Reflexivity was central throughout the study through piloting the topic guide with our Patient and Public Involvement and Engagement (PPIE) panel and reflecting on the experience, re‐listening to the original interviews and engaging in collaborative team discussions during theme development. The COREQ checklist was used to guide reporting^24^ (Appendix S1).

### 2.2 Recruitment and sample

To ensure a diverse participant pool, we purposively recruited participants who were employed as clinical pharmacists and sought to recruit from a variety of age, gender, sex and ethnicity. We also recruited from a range of geographical locations to ensure a diverse mix of clinical pharmacists working in urban and rural areas to understand if the role may differ across locations.

Our sample size was determined by the principle of information power, considering the study objectives, participant characteristics, anticipated data quality and analysis strategy.^25^ Based on these factors, we estimated recruiting approximately 10 clinical pharmacists.

Participants were recruited through GP practices, NHS networks, known contacts of the research team, social media and supplemented with snowball sampling methods. Ten GP practices and NHS networks were contacted by the research team or the Research Delivery Network (NIHR RDN), with the study details and a poster for them to distribute among their team/networks; interested potential participants were asked to contact the research team directly. Seventeen eligible potential participants self‐identified through these techniques and contacted the research team to take part in the study. In addition, we conducted a national survey of clinical pharmacists in 2022–2023 as a precursor to this study. We asked survey participants (*n* = 59) to register if they were interested in being interviewed. We followed up with these contacts by sending relevant recruitment materials to them. Upon expression of interest, potential participants were screened utilising the inclusion criteria and then received participation information sheets and were subsequently consented for the study. Recruitment started on 1 July 2022 and finished on 1 March 2023.

### 2.3 Ethical approvals

Ethical approval was obtained from the Health Research Authority (HRA) and Health and Care Research Wales (23/LO/0054) and University College London Research Ethics Committee (3344/006).

### 2.4 Inclusion criteria

- Work in primary care (e.g. general practice, community teams, care homes).
- Able to provide informed consent and had capacity.

### 2.5 Data collection

Interviews were guided by an interview schedule developed in collaboration with our PPIE panel drawing on the survey findings. The PPIE panel consisted of 2 family carers with lived experience of supporting someone with dementia whilst also receiving support from a clinical pharmacist. The interview schedule was piloted with our PPIE panel and refined before proceeding with interviews. Please see Appendix S2 for the full interview guide.

Interviews were conducted between December 2022 and March 2023 by telephone or video‐conferencing software (Microsoft Teams or Zoom). Informed written consent was obtained prior to the interviews. They were conducted by a member of the research team (A.W.), who is a female, postdoctoral researcher trained in qualitative research methods. Interviews lasted 30–60 min and were audio‐recorded and transcribed verbatim. Transcripts were checked against the recordings by a female research assistant (A.B.) to ensure accuracy.

Demographic information was collected from participants to contextualise the data and gauge if we were fulfilling our sampling strategy. Each participant completed a debrief form post‐interview to provide feedback about the interview along with demographic information. All study documents, including the questionnaire and feedback form were developed with support from our PPIE panel. A £20 shopping voucher was provided to participants in thanks (Appendix S3 contains the debrief form).

### 2.6 Data analysis

Transcripts were uploaded to NVivo 14^26^ and analysed using reflexive thematic analysis.^23^ The research team had a mix of professional backgrounds (GPs, pharmacists, social care and psychology) all of whom had experience in qualitative research. A.B. and N.D. coded 2 transcripts initially, then discussed and agreed on codes. Coding was discussed with the whole team and refined. A.B. coded all remaining interviews with regular discussions and input from N.D. and A.W. to refine and develop new codes. Themes were generated from the coding and discussed among the whole team, iteratively refined and defined. All authors provided feedback on codes and themes. Analysis was led by a research assistant trained in qualitative research methods with professional experience in dementia care and research (A.B.), and supervised by N.D., a professor of ageing research and expertise in qualitative research methods. Both have clinical and research experience of working with people with dementia.

## 3 RESULTS

### 3.1 Sample characteristics

A total of 76 participants were contacted to take part in the study, of whom 63 declined to take part or did not respond. Thirteen clinical pharmacists were interviewed one‐to‐one, of whom ten provided demographic information as well as feedback on the debrief form (see Table 1). Thirteen was agreed by the team as sufficient to answer our research question aligning with the principles of information power. We did not receive responses for demographic information and feedback from the remaining 3 participants. Of the 13 recruited, 10 were recruited from the precursor study, 2 from NHS networks and 1 from snowballing techniques.

**TABLE 1:** Demographic characteristics of 10 of 13 participants.

		Clinical pharmacist
Age in bands (years)	18–35	3
	36–45	1
	46–55	4
	56–65	1
	Not answered	1
Sex	Female	7
	Male	3
Ethnic group by UK Census categories	White—English	5
	Other White	1
	Indian	2
	Arab	1
	Pakistani	1
Education	Degree level	4
	Master's level	5
	Postgraduate Diploma	1
Professional Grade^a^	Band 6	4
	Band 7	1
	Band 8	5
Time working as clinical pharmacist (years)	<1	1
	2–3	1
	>5	8
Experience in primary care (years)	<1	1
	2–3	3
	4–5	1
	>5	5
Work location	London	3
	South West England	1
	Yorkshire and Humber	2
	South East England	1
	North West England	3

^a^ Positions in the NHS come with a pay band other than doctors, dentists and senior managers who have their own pay scales. The band determines the level of pay for the job role and contains sub‐bands. The higher the band, the more experienced or specialised the role.^27^ See https://www.nhsemployers.org/publications/nhs‐terms‐and‐conditions‐pay‐poster‐202223

Three overarching themes, each with subthemes, were developed from the data in interviews: (i) holistic care beyond medication management; (ii) the integral role and capacity of clinical pharmacists in the healthcare team and system when delivering dementia care; and (iii) building expertise in dementia care and defining the role of a clinical pharmacist.

### 3.2 Holistic care beyond medication management

Participants mentioned that one of their main responsibilities within the role is to review medications and undertake structured medication reviews with people with dementia. This often involved deprescribing to address potential polypharmacy, reduce medication‐related side effects and rectify instances of inappropriate prescribing to enhance safe and effective delivery of care for people with dementia:

“A lot of my role is speaking to patients about their medication […] I suppose one of the things we're focusing on is deprescribing where the main concern is, are patients on medications that they don't necessarily need. Especially dementia patients in particular, are they on medications that are giving them side effects, (or) is it medications that they keep ordering every month, but they don't really take?” [CP04]

Clinical pharmacists undertake reviews to ensure medication optimisation (which includes medication management and adherence), however participants discussed how they felt a duty of care that moved beyond medications. Several participants described their role as being part of a larger team working alongside other stakeholders taking a holistic approach to assessing and supporting people with dementia, not just the safe and effective use of medication:

“I think it's to be holistic, so don't just think about, do you take your meds, thank you very much, goodbye […] ask about falls, about day‐to‐day and what could be better, think about MDT [multidisciplinary team] involvement, where the patient might be struggling with things like isolation, finances, or cooking for themselves.” [CP03]

Some actively provided signposting to nonclinical support by facilitating referrals to social prescribers based in primary care and other local community‐based services such as health and wellbeing services, day centres and allied health professionals (e.g. physiotherapists), illustrating a holistic care approach and their wider care remit:

“A lot of elderly patients with polypharmacy who are struggling with social aspects, perhaps housing. So, we've done a lot of social prescriber referrals of health and wellbeing coaches for people struggling with housing and mould in their houses, who need advocates who need to help them with what to do next and how to do documents and forms etc.” [CP07]

Some considered it important to take a holistic approach proactively, as part of rigorous and thorough medication optimisation. Participants discussed how this involves recognising environmental changes, signs such as mouldy food at home and other indicators that warrant attention during home visits for people with dementia. This varied among participants possibly due to reasons such as experience and confidence in role and with the patient group:

“Look out for the red flags and actually, have a look: is the food in the kitchen looking fresh? Is there a smell of urine in the place? What does the state of—is their leg swollen? You know, it's the red flags.” [CP01]

Participants considered that a clear part of their role was their ability to see patients in a variety of settings, including at home. Home visits enabled them to adopt a holistic and proactive approach, promoting comprehensive assessments, where they gained insight into medication adherence as well as abilities to manage activities of daily living. This provided a more complete picture of a patient's overall wellbeing and identify red flags. There were some barriers to this holistic approach from remote (online or telephone) appointments:

“I think because we tend to do things remotely at the moment as well, I think that is another issue. Prior to Covid, if I was doing a big polypharmacy review, I would go out and see the person [with dementia] and that would give me the chance to look through their medicine cabinet, check what they've got, what they haven't got, and how it looks like they're coping.” [CP08]

However, some clinical pharmacists noted challenges with supporting people with dementia who are living at home and may not have support from a carer. It can be a challenge for clinical pharmacists when changing medications or introducing new tablets for those who do not have regular support or visits from family members:

“I think that's the struggle with these patients because when they've got dementia, either they're alone and there is no carer and I think that's where the problem lies. Or they've got kids who visit once, twice, 3 times a week. They're not there managing their medications. I think it becomes really difficult once the medication changes more than anything.” [CP11]

### 3.3 The integral role and capacity of clinical pharmacists in the healthcare team and system when delivering dementia care

Clinical pharmacists can have an integral role in the MDT, using their expertise to provide support to GPs and other colleagues about medications and administration in dementia care. The system of MDT working was considered supportive and effective, for both the primary care team and patients, in providing proactive and holistic patient care. MDT meetings provided opportunities to link with different organisations and specialities, enabling quick responses to questions and facilitating better care coordination for people with dementia:

**“**We hold an MDT, which is fantastic because we'll have a consultant geriatrician, a person from the care home, a dietician, a physiotherapist and somebody from mental health for older people, and I attend those as a pharmacist, so then we can truly holistically review a [care home] resident who's been referred in because of some difficulty or challenge.” [CP02]

Several participants thought they had more time than other healthcare professionals, such as GPs, to provide assistance with medications and other queries during appointments. They used this time to discuss medications, describing their purposes and potential side effects, ensuring patients have a comprehensive understanding, as well as other aspects related to patient health:

“So even when we're prescribing a repeat medication, we're not just going to sign it and tick it off sort of thing. And every GP I've worked with, they admit to this, but they've got hundreds to sign and so they do, and they're just like, “It's repeat, it's authorised, they're up‐to‐date, off they go’”. But we will look at the bloods, we will look at everything to make sure. So I think we've got that extra vigilance as well when it comes to managing these patients [people with dementia] if something is due or if something doesn't seem right.” [CP11]

While the MDT approach offered numerous benefits for maximising patient care and response efficiency, challenges were noted, particularly regarding deprescribing in people with dementia. Participants noted instances where some GPs were hesitant to deprescribe medications that may be contributing to cognitive decline due to concerns about potential negative outcomes:

“It's harder, I think a lot of GPs are afraid to deprescribe; what if the patient comes to harm? Whereas I would like them to be a little bit bolder sometimes and say, look, let's remove this in that we think there is a chance of worsening their dementia, or even causing dementia, and use an alternative.” [CP02]

Moreover, poor communication within a GP surgery between nurses, GPs and pharmacists, and an unsupportive primary care team presented major challenges to supporting people with dementia in their role:

“[ … challenges include] poor communication within the surgery, stuff like that, if you've not got a good vibe going on and one team is not talking to another […] or if the pharmacist hasn't got a particularly good relationship with the GP or nurse.” [CP04]

### 3.4 Building expertise in dementia care and defining the role of a clinical pharmacist

Dementia training beyond the Centre for Pharmacy Postgraduate Education (CPPE 1) pathways^28^ is currently optional. While some participants expressed interest in and pursued such training, others found it difficult to give the time to attend courses, particularly during breaks, or local training sessions were unsuitable:

“I wouldn't have had the training I had unless I was working out of that location; I would never have had that level of training, you know, by attending CPPE. I've been on dementia courses before, and this was at another scale.” [CP06]

Most participants had followed CPPE pathways, that typically include one module on dementia. Consequently, participants had to proactively seek additional training courses or research opportunities. This approach, however, varied among different locations. Some mentioned a lack of in‐depth readily available dementia‐specific training, and felt that they needed to rely on colleagues' expertise to develop their confidence and understanding of dementia care:

“I did the CPPE Medicine Optimisation in Care Home training and there was a whole module on that about dementia. Other than that, I tend to just pick up bits as we go along really. We have a senior consultant pharmacist, and she would perhaps do a little session on dementia. So probably not an awful lot really [of dementia training].” [CP08]

Autonomy played a significant role in charting one's own course in training and specialisation in dementia, and this varied among those interviewed:

“So, I think it's important for some autonomy of all clinicians, including pharmacists, for example, if I had a supervisor and I say, I want to learn more about dementia, I want to review them a little bit more or to have the space to book in a couple of patients to then discuss and reflect on. And then you're truly increasing your scope of practice but also being able to support a broader range of patients.” [CP03]

Participants new in the role needed time to feel self‐assured and confident in their responsibilities, particularly supporting and communicating with patients with cognitive impairment. Despite receiving training, their confidence in applying what they had learned, including care for people with dementia, varied. They considered that some clinical pharmacists might not be implementing medication changes in people with dementia, potentially due to inexperience and lack of confidence in altering patient care:

“I think, generally speaking, especially if you're talking maybe dementia patients and if they've got a lot of other issues, there they would be thought of as a relatively complex case patient and I think maybe some pharmacists, it depends, they may not have too much to do with that or they might just, sort of, tinker around the sides rather than make any sort of significant changes.” [CP04]

Moreover, with the role of clinical pharmacists being relatively new, participants felt they frequently needed to explain and clarify it to both people with dementia and their families. Participants reported a lack of understanding from patients about where clinical pharmacists fit in the wider health care service, often confusing them for community pharmacists at their local pharmacy or chemist:

“I think one of the main challenges that we have, which is ridiculous because it comes up all the time, is that people don't really understand where we fit in. So, I don't think our role is established enough or well known about and I think people often think we are the community pharmacist. So, they'll start asking us about when they can come and collect their [medication].” [CP08]

Despite these challenges and the need to establish a professional identity different from the traditional community pharmacist, when asked to describe their role to a patient, such as a person with dementia or their carer, many simplify this as ‘helping with medication’. They reframed their role to focus on elements that patients may understand. This included omitting to explain their role extends beyond medication management to include referrals and providing social support related to dementia. Some gave examples of how they balanced the information provided to patients by not being overly inclusive and confusing but giving enough information to demonstrate how they differ from a community pharmacist:

“I usually say I'm a pharmacist, so I'm a chemist, because that's how we used to be called. It's usually explained in quite loose terms but it's usually just we're dealing with medication, I am a chemist, but I don't work in a chemist shop, I work in the GP practice so I help the doctor with some of the medication issues.” [CP09]

## 4 DISCUSSION

### 4.1 Summary of main findings

This paper explores the experiences of clinical pharmacists when supporting people with dementia in primary care, as well as their responsibilities and requirements. Clinical pharmacists' activities are wide‐ranging, extending beyond traditional medication management to include holistic support, addressing the broader needs of people with dementia. Many pharmacists leveraged their expertise to provide comprehensive care, including coordination with MDT colleagues and proactively engaging in home visits, to promote overall patient wellbeing. However, dementia added an additional layer of complexity when clinical pharmacists were involved in managing medications. Supporting people with dementia requires specific skills and knowledge and pharmacists often highlighted many barriers for their colleagues to access opportunities to develop these. Subsequently, this led to variability in the support and services provided by clinical pharmacists for people with dementia.

### 4.2 Link to existing literature

Clinical pharmacy services are not limited to medication management. The pharmacists in our research frequently highlighted their role in holistic care provision. A recent scoping review found that while clinical pharmacists were involved with holistic medication management services, they generally focused on achieving clinical and economic outcomes and preventing drug‐related problems.^29^ The authors emphasised the importance of prioritising patient satisfaction, alongside these other outcomes, when conducting holistic patient reviews.^29^

Our study highlighted that pharmacists considered proactive and holistic reviews provided numerous benefits to people with dementia, particularly when undertaken in the home. Research has shown that proactive medication‐related recommendations made by pharmacists with appropriate experience and training, are widely accepted by other clinicians.^30^ There is wide consensus about the importance of proactive care for people living with MLTCs, including dementia. The shift from a reactive, episodic and fragmented approach in primary care settings can improve health outcomes including quality of life^31^ and mitigate the negative impact of frailty on individuals and healthcare systems.^32^

With increasing participation of clinical pharmacists in primary care teams, many studies have highlighted their critical role in enhancing efficacy of patient care and medication safety for people with dementia, providing reliable medication expertise and recommendations for prescribing and deprescribing.^33^, ^34^ However, pharmacists can face a diverse range of barriers when supporting medication management and optimisation in people with dementia, including challenges with interprofessional communication and collaboration^34^ and adherence,^35^ as also identified in our research. Training and practice autonomy disparities were also identified barriers that led to variations in expertise among clinical pharmacists and may impact their ability to implement medication changes for people with dementia without further support from the MDT. Our study suggests that clinical pharmacists can help optimise medication and address broader health needs holistically, but importantly patient outcomes, including people with dementia, may be enhanced when pharmacists are working and communicating closely with GPs and other healthcare professionals in a supportive environment.

By empowering and equipping clinical pharmacists with the right skills and encouraging a supportive team environment, they can contribute to dementia care in the MDT successfully. A recent review has shown communication problems are a barrier in implementing deprescribing and medication changes.^36^ Enabling factors included an MDT approach, improved communication, clinician education and regular medication reviews.^36^ Healthcare professional training which focuses on working as part of the MDT and involving patients in shared decision making has been suggested as helping the deprescribing process.^37^

### 4.3 Implications for policy and practice

Future policy should seek to increase clinical pharmacists' confidence and skills on successful communication with fellow health and care professionals and people with dementia.^38^ Training courses should be readily available nationwide to ensure a standardised approach to dementia care and should include real‐world examples, practice‐based learning and focus on developing transferable skills, including communication and confidence, that pharmacists can apply when working in a team and supporting patients.^39^

Enhancing public awareness of the accessibility as well as the usefulness of clinical pharmacists in supporting patients in primary care, including people with dementia, such as their ability to review and optimise medication to reduce polypharmacy, would be beneficial.

### 4.4 Strengths and limitations

To our knowledge, this is the first study exploring clinical pharmacists' experience of supporting people with dementia in primary care. The main strength is the use of semistructured interviews that enabled participants to describe in detail their work as well as challenges. Moreover, our sample was diverse and fulfilled our purposive sampling framework.

The small study is, however, limited by participation of clinical pharmacists only working in England and, although we sampled a diverse range of participants from experience to location, results may not be transferable to other countries in the UK or worldwide. Clinical pharmacists are embedded throughout the 4 nations of the UK and future studies should cover all 4 nations. However, this is one of the first studies to explore the role of clinical pharmacists within primary care to support people with dementia.^20^ Our aim was fairly narrow and the quality of the data was high with rich content, which aligning with the concepts of information power would suggest our sample is sufficient to answer the research question.^25^ The views of other MDT members, patients and carers would provide further insights.

## 5 CONCLUSION

Clinical pharmacists provide wide‐ranging support beyond medication management, including holistic patient care and MDT collaboration. They provide proactive support assessing patient wellbeing, including through home visits. This study highlights the importance of confidence, training and a supportive environment for clinical pharmacists to effectively contribute to dementia care.

## AUTHOR CONTRIBUTIONS

A.B. performed the analysis and wrote the manuscript. A.W. conducted the interviews. N.D. was PI and secured funding for the project, and supported the analysis and writing of the manuscript. K.B., C.B., Y.J., J.M., M.O., G.R., M.S., K.S., V.V., J.W. and J.W. read and made edits to the manuscript.

## CONFLICT OF INTEREST STATEMENT

The authors declare they have no competing interests.

Ethical approval was obtained from the Health Research Authority (HRA) and Health and Care Research Wales (23/LO/0054) and University College London Research Ethics Committee (3344/006).

## Supporting information


**APPENDIX S1** Supporting Information

**APPENDIX S2** Supporting Information

**APPENDIX S3** Supporting Information

## Data Availability

Data sharing is not applicable to this article as no datasets were generated or analysed during the current study.

## Footnotes

1 CPPE is contracted by Health Education England to provide training to primary care pharmacists by offering training pathways to align the education requirements of pharmacists and pharmacy technicians.^28^ See https://www.cppe.ac.uk/career/pcpep/pcpep-training-pathway
