# Supplementary material for: The potential for clinical pharmacists to support older people with dementia in the community: A qualitative interview study
Source: Br J Clin Pharmacol. 2025 Jul 8;91(11):3141–9. doi: 10.1002/bcp.70160 (PMC12569555; doi:10.1002/bcp.70160)
Supplement: Supplementary file 3 — APPENDIX S3 Supporting Information [file BCP-91-3141-s001.docx]

# About you

| Age | | years | |
| --- | --- | --- | --- |
| Gender | | Male  Female  Prefer not to say | |
| Ethnicity | **White**  English / Welsh / Scottish / Northern Irish / British  Irish  Gypsy or Irish Traveller  Any other White background  **Mixed / Multiple ethnic groups**  White and Black Caribbean  White and Black African  White and Asian  Any other Mixed / Multiple ethnic background  **Asian / Asian British**  Indian  Pakistani  Bangladeshi  Chinese  Any other Asian background | | **Black / African / Caribbean / Black British**  African  Caribbean  Any other Black / African / Caribbean background  **Other ethnic group**  Arab  Any other ethnic group |
| Main language spoken | English  Panjabi  Gujarati  Chinese  Welsh  Urdu  Arabic  Portuguese  Polish  Bengali  French  Spanish  Other: specify: | | |
| Level of education: | Degree  Masters level degree  PhD  Other: specify: | | |
| Please state your professional grade | Band 6  Band 8  Band 7  Band 9  Other: Specify: | | |
| What is your current job title? |  | | |
| How many years experience do you have as a clinical pharmacist overall? | Less than 1 year  4-5 years  1-2 years  More than 5 years  2-3 years | | |
| How many years experience do you have as a clinical pharmacist in general practice? | Less than 1 year  4-5 years  1-2 years  More than 5 years  2-3 years | | |
| What is the location in which you work? | North East  South West  North West  London  Yorkshire and the Humber  South East  East Midlands  Scotland  West Midlands  Northern Ireland  East of England  Wales | | |
| Which best describes the location you work in? | Urban  Rural  Semi-rural | | |

**Interview feedback**

We would like to take the opportunity to thank you for completing this interview. *Without you, this research would not be possible.* We would be grateful if you could provide some feedback on your experience of completing the interview:

| Do you feel you could cope with the length of the interview? | Yes, quite easily | Only just | No |
| --- | --- | --- | --- |
| Did you find taking part in the study helpful? | Yes, very | A little helpful | No |
| Did you feel the study caused you distress? | Yes, a lot | A little | No |
| Do you have any other comments about taking part in this study? |  | | |

*Source: Sque, M., Walker, W., & Long-Sutehall, T. (2014). Research with bereaved families: a framework for ethical decision-making. Nurs Ethics, 21(8), 946-955.*

| Would you like us to send you any further written information on any of the topics raised today? | Yes  No  If yes, please specify: ____________________________  ______________________________________________ |
| --- | --- |
| When the study is completed, would you like to receive a written summary of the study findings? | Yes  No |
| Do you have any further questions about the study or how the findings will be used? |  |

**Thank you**

Any data you have provided today will remain both confidential and anonymous and will be used only for the purposes outlined here. Participation in the study is voluntary and you may withdraw your assistance at any time if you wish and without explanation. You may use the contact number below should any queries or concerns arise in the future.

**Thank you for your participation.**

**Dr Nathan Davies, Chief Investigator**

**Tel: 020 3108 6616; E-mail:** [**n.m.davies@ucl.ac.uk**](mailto:n.m.davies@ucl.ac.uk)
